# Assessing the comparability of cycle threshold values derived from five external quality assessment rounds for omicron nucleic acid testing

**DOI:** 10.1186/s12985-023-02032-z

**Published:** 2023-06-08

**Authors:** Gaowei Fan, Yali Jin, Qingtao Wang, Yuhong Yue

**Affiliations:** 1 Department of Clinical Laboratory, Beijing Chao-Yang Hospital, Capital Medical University, 8 Gongren Tiyuchang Nanlu, Chaoyang District, Beijing, China; 2 Beijing Center for Clinical Laboratory, Beijing, China; 3 Beijing Medical Laboratory Quality Control and Improvement Center, Beijing, China

**Keywords:** SARS-CoV-2, Omicron, RT-PCR, Cycle threshold, Qualitative test, Comparability

## Abstract

**Background:**

A variety of open-system real-time reverse transcriptase polymerase chain reaction (RT-PCR) assays for several acute respiratory syndrome coronavirus 2 are currently in use. This study aimed to ensure the quality of omicron nucleic acid testing and to assess the comparability of cycle threshold (Ct) values derived from RT-PCR.

**Methods:**

Five external quality assessment (EQA) rounds using the omicron virus-like particles were organized between February 2022 and June 2022.

**Results:**

A total of 1401 qualitative EQA reports have been collected. The overall positive percentage agreement was 99.72%, the negative percentage agreement was 99.75%, and the percent agreement was 99.73%. This study observed a significant variance in Ct values derived from different test systems. There was a wide heterogeneity in PCR efficiency among different RT-PCR kits and inter-laboratories.

**Conclusion:**

There was strong concordance among laboratories performing qualitative omicron nucleic acid testing. Ct values from qualitative RT-PCR tests should not be used for clinical or epidemiological decision-making to avoid the potential for misinterpretation of the results.

**Supplementary Information:**

The online version contains supplementary material available at 10.1186/s12985-023-02032-z.

## Background

A new severe acute respiratory syndrome coronavirus 2 (SARS-CoV-2) variant of concern, omicron, increased rapidly since its emergence and caused another wave of infection [1]. Access to quality-assured diagnostic assays for omicron is essential for curtailing the spread of coronavirus disease (COVID-19) [2, 3].

Dozens of assays have been emergency approved by China National Medical Products Administration (NMPA) for SARS-CoV-2 diagnosis, and nucleic acid testing by real-time reverse-transcription polymerase chain reaction (RT-PCR) is the mainstay of COVID-19 diagnosis [4, 5]. These RT-PCR kits provide a qualitative result along with cycle threshold (Ct) values. Published studies found that the Ct values of the SARS-CoV-2 target region change based on the infection stage and sometimes are interpreted as semiquantitative [6, 7]. Thus, the Ct values have been used to assess viral load [3, 8], infection [9], infection severity [7, 10], and in determining quarantine measures [7]. Patients with high Ct values in the late infection stage seemed no longer infectious [11, 12]. A combination of Ct values and infection stage might shorten the isolation period, reducing the burden on healthcare infrastructure [7, 13].

Most of the COVID-19 testing laboratories in China use open-system RT-PCR assays composed of different kits and instruments [14, 15]. The modified assays should be appropriately validated before use. Ct values are affected by all aspects of SASR-CoV-2 testing, including specimen sampling, processing, nucleic acid extraction, reverse transcription, amplification, and data analysis [16–19]. The increased use of SARS-CoV-2 Ct values makes comparability of Ct values essential [11].

External quality assessment (EQA) is essential for ensuring reliable test results and helps assess Ct values' comparability. To clarify the detection ability for omicron and to assess the comparability of Ct values derived from RT-PCR, five EQA rounds were conducted between February 2022 and June 2022 in Beijing, China.

## Materials

### Preparation of SARS-CoV-2 virus-like particles

SARS-CoV-2 virus-like particles (VLPs) were constructed using armored RNA enveloping technology [20, 21]. Briefly, the sequence of omicron was from the GISAID data set. The backbone sequence Wuhan-Hu-1 (GENBANK accession number NC_045512.2) was modified by containing omicron (BA.1) mutation. The targeted sequences in the ORF1ab, N, and E genes were synthesized and cloned into the expression vector and were then transformed into the *Escherichia coli* strain for VLPs expression. The cells were harvested and lysed, and the VLPs were purified by gel exclusion chromatography. To eliminate the synthesized DNA, the VLPs were incubated with DNase I. A QX200 droplet digital PCR (BioRad) was utilized for quantification.

The omicron VLPs were diluted into 2.0 × 10^3^, 1.0 × 10^3^, 5.0 × 10^2^, and 2.0 × 10^2^ copies/mL using virus preservation solution and were tested by 15 commercial SARS-CoV-2 RT-PCR kits from DaAn Gene Co., Ltd, referred to as DaAn; Shanghai BioGerm Medical Technology Co., Ltd, referred to as BioGerm; Beijing Nagene Diagnosis Reagent Co., Ltd, referred to as Nagene; Wuhan EasyDiagnosis Biomedicine Co., Ltd, referred to as EasyDiagnosis; Jiangsu Bioperfectus Technologies Co., Ltd, referred to as Bioperfectus; Sansure Biotech Inc., referred to as Sansure; Zybio lnc., referred to as Zybio; Shanghai Geneodx Biotechnology Co., Ltd, referred to as Geneodx; Beijing Kinghawk Pharmaceutical Co., Ltd, referred to as Kinghawk; Guangdong Hybribio Biotech Co., Ltd, referred to as Hybribio; Beijing Applied Biological Technologies Co., Ltd, referred to as ABT; ﻿Maccura Biotechnology Co., Ltd, referred to as Maccura; Shanghai Zhijiang Biotechnology Co., Ltd, referred to as Zhijiang; Shanghai Fosun Pharmaceutical (Group) Co., Ltd, referred to as Fosun; and ﻿BGI Bio-tech Co., Ltd, referred to as BGI.

### Homogeneity and stability evaluation

Homogeneity evaluation was conducted according to the CNAS-GL003:2018 Guidance [22]. Briefly, the sample was diluted into concentrations of 4.0 × 10^3^, 2.0 × 10^3^, 1.0 × 10^3^, 7.5 × 10^2^, 5.0 × 10^2^, 2.5 × 10^2^ and 2.0 × 10^2^ copies/mL. Then, the dilutions were aliquoted and stored at -20 °C. Ten samples of each concentration were randomly selected for RNA extraction and were tested in triplicate. The Ct values of the ORF1ab and N gene were analyzed using a one-way analysis of variance (ANOVA). Short-time stability study was conducted to assess the stability of the VLPs during delivery under cold chain conditions. The omicron VLPs were stored at 2–8 °C for various times (1, 5, 10 days). After that, all the samples were tested in triplicate, and two independent t-test were performed.

### Organization of EQA

Five EQA rounds were conducted using omicron VLPs between February 2022 and June 2022 in Beijing, China. The EQA program was accredited to the ISO/IEC 17043. Each EQA panel consisted of five or six coded samples, two were negative, and the rest three or four were omicron positive. The positive EQA samples were at the concentration of 2.0 × 10^2^–2.0 × 10^3^ copies/mL.

The laboratories performing SARS-CoV-2 nucleic acid testing were asked to participate in the EQA schemes. The EQA panels were transported to the laboratories under cold chain conditions. The participants were asked to test the EQA samples using their routine molecular assay. The qualitative interpretation of the EQA results associated with other assay run data, such as Ct values, nucleic acid extraction kits, RT-PCR kits, and PCR instruments, were asked to submit through an online reporting system (http://corelab.clinet.com.cn/) within a 2-day time window upon receiving the EQA panels. The qualitative interpretation of the EQA data was scored, and a laboratory that correctly reported all the EQA samples was classified as competent.

### Statistical analysis

Categorical data were represented as counts and percentages, and numerical data were reported as median (interquartile range, IQR) or mean (standard deviation, SD).

The proportion of competent laboratories, the positive percentage agreement (PPA), the negative percentage agreement (NPA), and the percent agreement were calculated. SD was adopted to assess the diversity of Ct values derived from RT-PCR. Ct values determined by a pre-amplification step were excluded.

Linear regression based on the Ct value versus log copy was performed by the following equations [23]:

$${\text{Ct}} = a + blog_{10} c,$$

$$E = 10^{{ - \left( {1/b} \right)}} - 1,$$

where *a* is the intercept, *b* is the slope, *c* is the concentration, and *E* is the amplification efficiency. The regression lines with the coefficient of determination (r^2^) < 0.94 were excluded [24].

Statistical analyses were performed by Chi-square test, Kruskal–Wallis test, Wilcoxon signed-rank test, one-way analysis of variance (ANOVA), and t-tests using GraphPad Prism 8 (GraphPad Software Inc., San Diego, CA, USA). *P* < 0.05 was considered significant.

## Results

### Evaluation of the omicron VLPs

The omicron VLPs with a concentration of 2.0 × 10^3^, 1.0 × 10^3^, 5.0 × 10^2^, and 2.0 × 10^2^ copies/mL were tested in duplicate by 15 commercial RT-PCR kits. All the RT-PCR kits reported correct qualitative results. Of them, 14 commercial RT-PCR kits can successfully detect the target genes. Zhijiang RT-PCR kit could only detect N and E genes but failed to detect ORF1ab because the VLPs didn’t contain the targeted sequence in the ORF1ab gene. According to the manufacturer’s instructions, the ORF1ab target failure did not influence the qualitative interpretation of the results.

Homogeneity evaluation showed no significant difference in Ct values among samples with the same concentration. The short-time stability study revealed that the EQA samples were stable under 2–8 °C for 10 days.

### Performance of the laboratories for the qualitative interpretation of EQA data

A total of 8116 EQA panels were collected. All the panels were detected using commercial SARS-CoV-2 RT-PCR assays. For each EQA round, the proportion of competent laboratories ranged from 98.55 to 99.63%, PPA ranged from 99.31 to 99.91%, NPA ranged from 99.31 to 100%, and percent agreement ranged from 99.31 to 99.94% (Table 1). The overall proportion of competent laboratories, PPA, NPA, and percent agreement was 99.14% (1389/1401), 99.72% (5299/5314), 99.75% (2795/2802), and 99.73% (8094/8116), respectively.

**Table 1:** The performance of the laboratories for the qualitative interpretation of EQA data

Panel ID	No. of labs	The proportion of competent labs	PPA	NPA	Percent agreement
202202	278	99.28% (276/278)	99.82% (1110/1112)	99.82% (555/556)	99.82% (1665/1668)
202203	273	99.63% (272/273)	99.91% (1091/1092)	100% (546/546)	99.94% (1637/1638)
202204	275	98.55% (271/275)	99.82% (1098/1100)	99.64% (548/550)	99.76% (1646/1650)
202205	285	98.95% (282/285)	99.65% (1136/1140)	100% (570/570)	99.77% (1706/1710)
202206	290	99.31% (288/290)	99.31% (864/870)	99.31% (576/580)	99.31% (1440/1450)
Overall	1401	99.14% (1389/1401)	99.72% (5299/5314)	99.75% (2795/2802)	99.73% (8094/8116)

EQA, external quality assessment; No., number; PPA, the positive percentage agreement; NPA, the negative percentage agreement

This study noted that 22 incorrect EQA results, namely, 8 false negative results, 2 false positive results, and 12 invalidated results, were reported. Further analysis showed that incorrect data entry by the participants (leading to 12 invalidated reports and one false positive report), problems with the test system (leading to 6 false negative reports), and problems associated with techniques such as sample mixed up and improper handling of the sample (leading to two false negative reports and one false positive report) were the cause of the incorrect results.

### Assessing the comparability in Ct values derived from RT-PCR

The EQA samples were tested by different extraction kits, RT-PCR kits, and PCR instruments. Ct values of the EQA samples with the same concentration were grouped to assess the comparability in Ct values. During the analysis, results containing clear outlier Ct values were excluded. As shown in Table 2, there was extreme variability in the Ct values for both ORF1ab and N. Regardless of the gene targets, the range of Ct values can be as large as 18 cycles. The IQR of the Ct values was 3 and 2 cycles for ORF1ab and N, respectively. There were 1404 results (39.24%) with absolute deviation from the respective median values by > 1 cycle, 681 results (19.03%) by > 2 cycles, 321 results (8.97%) by > 3 cycles, 141 results (3.94%) by > 4 cycles for ORF1ab. For N gene, 1482 results (41.66%) yielded absolute deviation from the respective median values by > 1 cycle, 704 results (19.79%) by > 2 cycles, 273 results (7.68%) by > 3 cycles, and 111 results (3.12%) by > 4 cycles.

**Table 2:** The Ct values for EQA samples were determined using various test systems for ORF1ab and N genes at different concentrations

Target gene	Concentration (copies/mL)	Number	Mean (SD)	Median (IQR)	Range	Skewness	Kurtosis
ORF1ab	2.0 × 10^3^	945	32.39 (2.104)	32 (31–34)	18	− 0.6157	3.574
	1.0 × 10^3^	942	33.35 (2.183)	33 (32–35)	18	− 0.932	4.378
	5.0 × 10^2^	939	34.36 (2.021)	34 (33–36)	15	− 0.4432	1.51
	2.0 × 10^2^	752	35.49 (1.979)	36 (34–37)	18	− 0.6895	2.859
N	2.0 × 10^3^	938	33.1 (2.179)	33 (32–34)	18	− 0.5439	2.25
	1.0 × 10^3^	936	33.97 (2.241)	34 (33–35)	18	− 0.8828	3.134
	5.0 × 10^2^	939	34.82 (2.112)	35 (34–36)	16	− 0.849	2.656
	2.0 × 10^2^	744	35.86 (1.904)	36 (35–37)	15	− 0.7259	2.336

Ct, cycle threshold; SD, standard deviation; IQR, interquartile range

### Assessing the comparability of Ct values determined by different RT-PCR kits

**Fig. 1:**
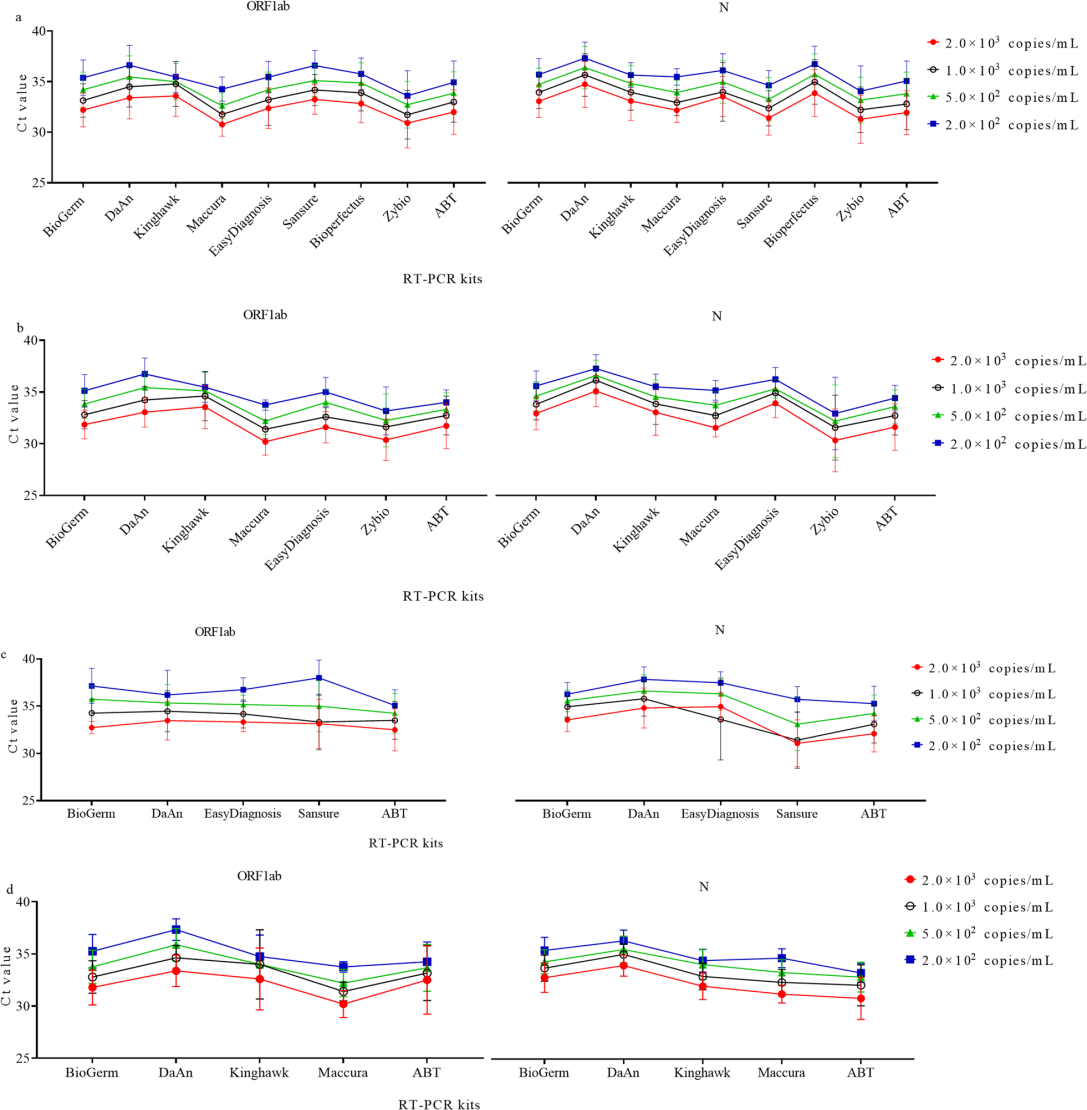
The Ct values derived from EQA samples were detected by different RT-PCR kits. **a** The Ct values determined by different RT-PCR kits combined with different extraction kits and different PCR instruments, **b** the Ct values determined by different RT-PCR kits combined with the Tianlong nucleic extraction kit and different PCR instruments, **c** the Ct values determined by different RT-PCR kits combined with the DaAn nucleic extraction kit and different PCR instruments, **d** the Ct values determined by different RT-PCR kits combined with Tianlong nucleic extraction kit and ABI7500 PCR instrument

There was a wide variation in Ct values obtained by different RT-PCR kits (Fig. 1a, Additional file 3: Table S1). The maximum SD was 2.55 cycles for ORF1ab and 2.93 cycles for N. There was a significant difference in Ct values among different RT-PCR kits. One should be noted that the comparison above did not consider the difference in nucleic acid extraction kits and PCR instruments.

To diminish the variation in nucleic acid extraction, we focused on the results determined by the same extraction kit combined with different RT-PCR kits and PCR instruments. For samples extracted by Tianlong Nucleic Acid Extraction kit (Tianlong Technology Co., Ltd), the SD ranged from 0.5 to 2.55 cycles for ORF1ab and 0.89 to 3.54 cycles for N when results were grouped by RT-PCR kits (Fig. 1b, Additional file 3: Table S1). The variation in Ct values reached statistical significance for ORF1ab and N among different RT-PCR kits. For samples extracted by DaAn Nucleic Acid Extraction Kit (DaAn Gene Co., Ltd), a significant difference in Ct values for the N gene was observed (Fig. 1c, Additional file 3: Table S1). The significant difference in Ct values for ORF1ab was only observed for samples of 2.0 × 10^2^ copies/mL. Noting that the statistical analysis did not consider the difference associated with PCR instruments and RT-PCR kits.

To avoid the diversity in extraction kits and PCR instruments, we focused on the samples detected by Tianlong Nucleic Acid Extraction kit and ABI7500 PCR instrument (Thermo Fisher Scientific, Waltham, MA, USA) (Fig. 1d, Additional file 3: Table S1). When grouped by RT-PCR kits, there was a significant difference in Ct values for ORF1ab for all concentrations except 5.0 × 10^2^ copies/mL. A significant variance in Ct values for N was observed among different RT-PCR kits. These findings indicated that different RT-PCR kits yielded less comparable Ct values.

### Assessing the comparability of Ct values obtained by different extraction methods

**Fig. 2:**
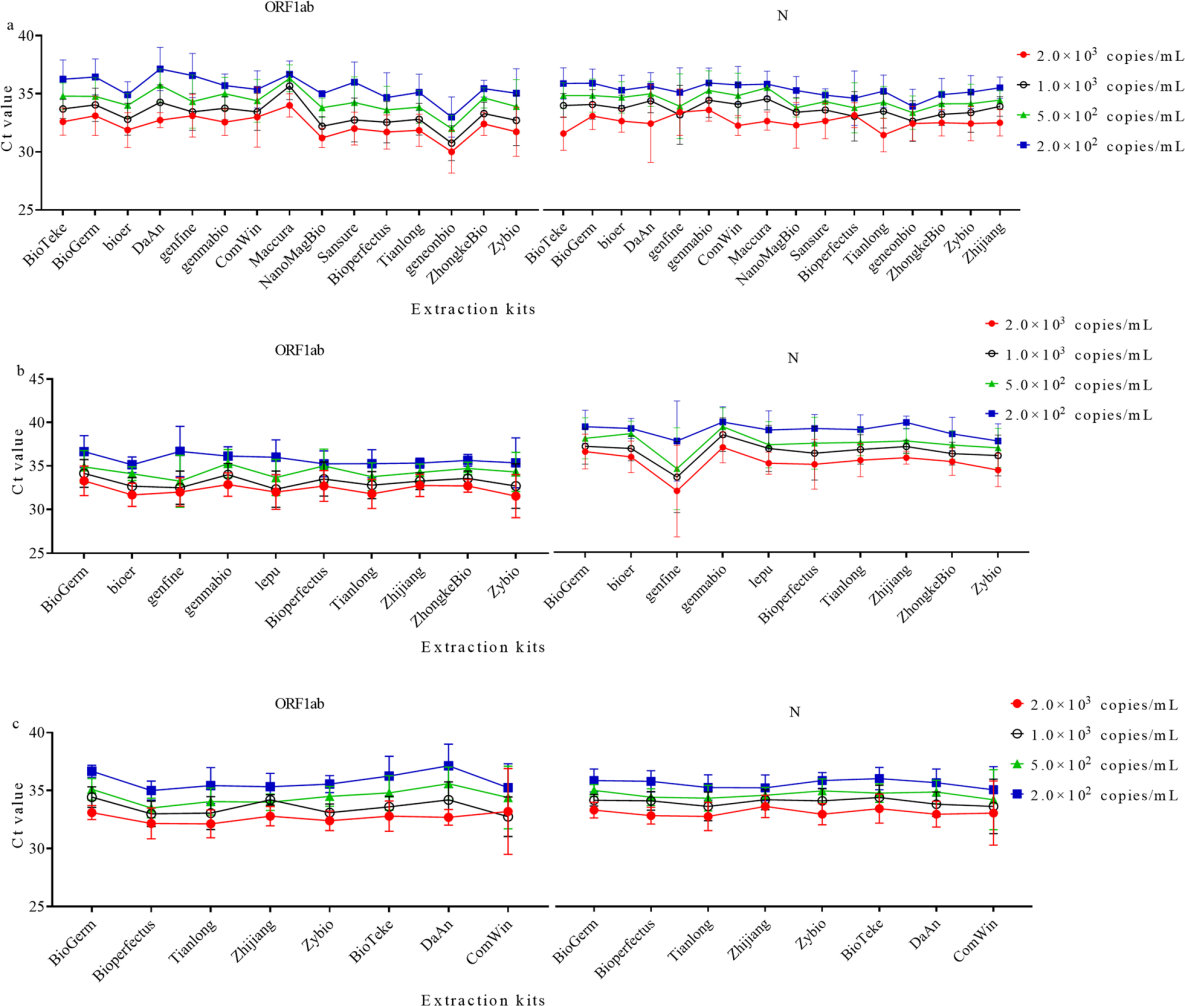
The Ct values derived from EQA samples were detected by different extraction kits. **a** The Ct values determined by different extraction kits combined with BioGerm RT-PCR kit and different PCR instruments, **b** the Ct values determined by different extraction kits combined with BioGerm RT-PCR kit and ABI7500 PCR instrument, **c** the Ct values determined by different extraction kits combined with BioGerm RT-PCR kit and SLAN PCR instrument

We analyzed the samples determined by the same RT-PCR kit but different extraction kits. For the samples tested by BioGerm RT-PCR kit, the SD ranged from 0 to 2.58 cycles for ORF1ab and 0 to 3.49 cycles for N gene across different extraction kits (Fig. 2a, Additional file 3: Table S2). There was a significant difference in Ct values for ORF1ab among different extraction kits. For N, the difference in Ct values reached significant for samples of 2.0 × 10^3^ copies/mL and 5.0 × 10^2^ copies/mL. The comparison above did not consider the variance in PCR instruments.

To avoid the effects of the diversity in PCR instruments, we compared the Ct values determined by test systems composed of BioGerm RT-PCR kit and ABI 7500 but different extraction kits (Fig. 2b, Additional file 3: Table S2). Among different extraction kits, a significant difference in Ct values was only observed for ORF1ab for the sample of 2.0 × 10^3^ copies/mL, there was no significant difference for N. For EQA samples tested by BioGerm RT-PCR kit and SLAN-96S/96P Real-Time PCR System (referred as SLAN PCR, Shanghai Hongshi Medical Technology Co., Ltd, China), a significant difference was observed for ORF1ab for samples of 2.0 × 10^3^, 1.0 × 10^3^, and 5.0 × 10^2^ copies/mL, and there is no significant difference for N when grouped by extraction kits (Fig. 2c, Additional file 3: Table S2). The findings indicated unlikely comparability in Ct values for ORF1ab among different extraction kits.

### Assessing the comparability of Ct values determined by different PCR instruments

**Fig. 3:**
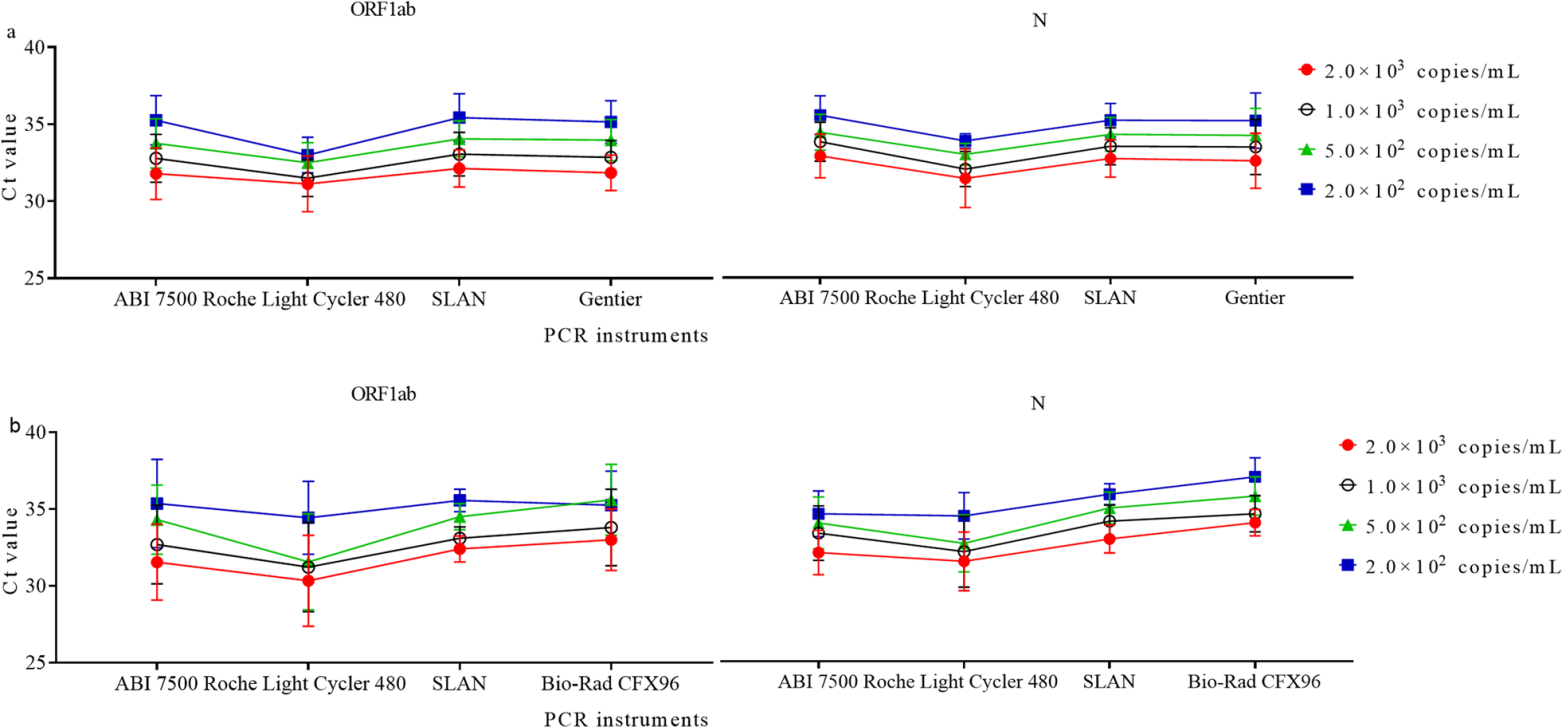
The Ct values derived from EQA samples were detected by different PCR instruments. **a** The Ct values determined by Tianlong nucleic extraction kit and BioGerm RT-PCR kit combined with different PCR instruments, **b** the Ct values determined by Zybio nucleic acid extraction kit and BioGerm RT-PCR kit combined with different PCR instruments

To assess the impact of the PCR instruments on the comparability of Ct values, we analyzed the results performed by the same extraction kits, the same RT-PCR kits but different PCR instruments. For samples tested by Tianlong Nucleic Acid Extraction kit and BioGerm RT-PCR kit, there was a significant difference in Ct values for ORF1ab for samples of 5.0 × 10^2^ and 2.0 × 10^2^ copies/mL among different PCR instruments (Fig. 3a, Additional file 3: Table S3). The difference in Ct values for N was statistically significant among different PCR instruments (Fig. 3a, Additional file 3: Table S3). Notably, the samples detected by Roche Light Cycler 480 Real-Time PCR System (referred as LC480, Roche Diagnostics, Mannheim, Germany) presented lower mean Ct values than those by other PCR instruments.

We also compared the Ct values by Zybio Nucleic Acid Extraction kit (Zybio lnc.) and BioGerm RT-PCR kit. Similarly, samples tested by LC480 presented the lowest mean Ct values. A significant difference in Ct values for ORF1ab for samples of 5.0 × 10^2^ copies/mL and N gene for samples of 2.0 × 10^3^, 5.0 × 10^2^, and 2.0 × 10^2^ copies/mL were observed (Fig. 3b, Additional file 3: Table S3). These results indicated that the difference in PCR instruments influenced the comparability of Ct values.

### Assessing the comparability of Ct values among different laboratories using the same test systems

**Fig. 4:**
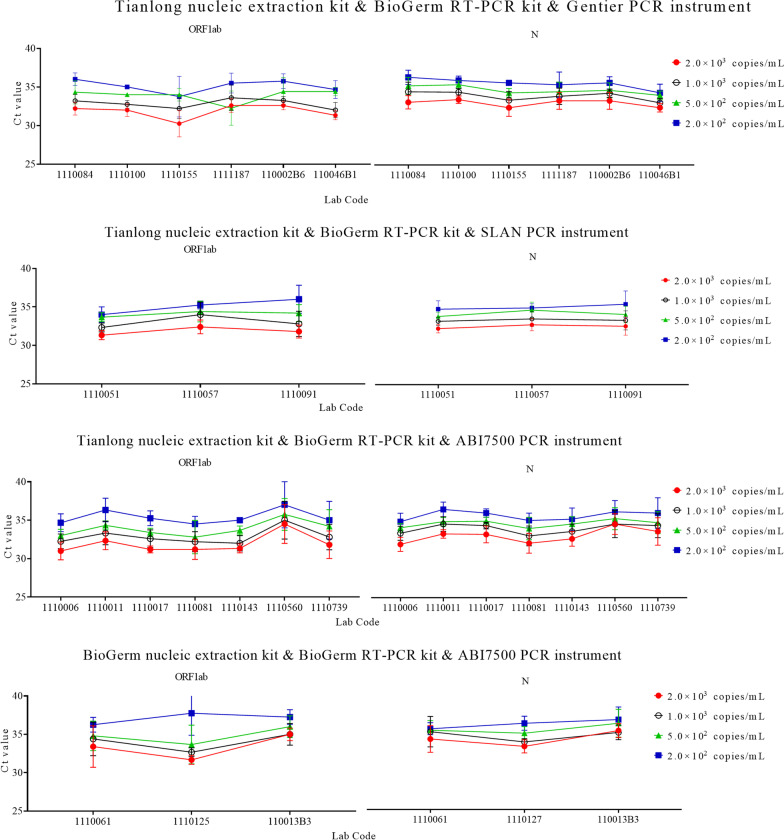
The Ct values derived from EQA samples were detected by different laboratories using the same test system

We then compared the Ct values among laboratories using the same test system. A laboratory offering less than 3 Ct values for EQA samples of the same concentration was excluded during the filtering stage. EQA results were determined by four different frequently used test systems, including Tianlong Nucleic Acid Extraction kit & BioGerm RT-PCR kit & Gentier 48E/48R/96E/96R Real-Time PCR System PCR instrument (referred as Gentier PCR, Tianlong Technology Co., Ltd), Tianlong Nucleic Acid Extraction kit & BioGerm RT-PCR kit & SLAN PCR instrument, Tianlong Nucleic Acid Extraction kit & BioGerm RT-PCR kit & ABI7500 PCR instrument, and the BioGerm Nucleic Acid Extraction kit (Shanghai BioGerm Medical Technology Co., Ltd) & BioGerm RT-PCR kit & ABI7500 PCR instrument were used for analysis (Fig. 4, Additional file 3: Table S4). No significant difference in Ct values for ORF1ab and N among laboratories using the same test system was found.

### Assessing the PCR efficiency through standard curves

To determine the suitable dilutions for standard curves, we used the Ct values for ORF1ab derived from the EQA samples with concentrations of 2.0 × 10^3^, 1.0 × 10^3^, 5.0 × 10^2^, and 2.0 × 10^2^ copies/mL. The EQA samples were determined by 12 different RT-PCR kits within a laboratory using the same extraction kit and PCR instrument (Table 3). r^2^ of the standard curves showed that all the RT-PCR kits except for BioGerm RT-PCR kit and NaGene RT-PCR kit had r^2^ > 0.94 when using four dilutions of 2.0 × 10^3^, 1.0 × 10^3^, 5.0 × 10^2^, and 2.0 × 10^2^ copies/mL (Table 3). When the samples of 2.0 × 10^2^ copies/mL were excluded, the r^2^ values for the 12 RT-PCR kits were > 0.95. As a result, three dilutions of 2.0 × 10^3^, 1.0 × 10^3^, and 5.0 × 10^2^ copies/mL were used for standard curves (Table 3). The amplification efficiency varied among different RT-PCR kits, with a minimum value of 61.55% and a maximum value of 128.24% (Table 3, Additional file 1: Figure S1).

**Table 3:** Calculation of PCR efficiency using ORF1ab Ct values for EQA samples

RT-PCR Kits	Three dilutions			Four dilutions		
	1/slope	E (%)	r^2^	1/slope	E (%)	r^2^
BioGerm	− 0.238	72.98	0.9913	− 0.3723	135.67	0.8586
DaAn	− 0.28	90.55	0.9902	− 0.2917	95.75	0.9956
BGI	− 0.2519	78.61	0.9614	− 0.2561	80.34	0.9863
Geneodx	− 0.2478	76.93	0.9995	− 0.2947	97.11	0.9824
Kinghawk	− 0.2083	61.55	0.9834	− 0.2641	83.70	0.9575
Hybribio	− 0.35	123.87	0.9682	− 0.4022	152.46	0.9753
﻿Maccura	− 0.3584	128.24	0.9618	− 0.3694	134.10	0.9858
EasyDiagnosis	− 0.3254	111.54	0.9839	− 0.3307	114.14	0.9943
Nagene	− 0.2213	66.46	0.9985	− 0.3253	111.49	0.9009
Sansure	− 0.329	113.30	0.9572	− 0.3277	112.67	0.9855
Bioperfectus	− 0.2951	97.29	0.9999	− 0.3609	129.56	0.976
Zybio	− 0.2676	85.18	0.9857	− 0.3531	125.48	0.9444

EQA, external quality assessment; r^2^, the coefficient of determination; E, efficiency; BioGerm, Shanghai BioGerm Medical Technology Co., Ltd; DaAn, DaAn Gene Co., Ltd; BGI, ﻿BGI Bio-tech Co., Ltd; Geneodx, Shanghai Geneodx Biotechnology Co., Ltd; Kinghawk, Beijing Kinghawk Pharmaceutical Co., Ltd; Hybribio, Guangdong Hybribio Biotech Co., Ltd; Maccura, ﻿Maccura Biotechnology Co., Ltd; EasyDiagnosis, Wuhan EasyDiagnosis Biomedicine Co., Ltd; Nagene, Beijing Nagene Diagnosis Reagent Co., Ltd; Sansure, Sansure Biotech Inc.; Bioperfectus, Jiangsu Bioperfectus Technologies Co., Ltd; Zybio, Zybio lnc

We also assessed the PCR efficiency of the participating laboratories. The Ct values from EQA samples with concentrations of 2.0 × 10^3^, 1.0 × 10^3^, and 5.0 × 10^2^ copies/mL were used. Only laboratories that reported at least three Ct values for EQA samples of the same concentration were included to avoid the random effects in each run [23]. The mean Ct values of the same concentrations versus the logarithm of the corresponding target concentrations were plotted onto the standard curves (Additional file 2: Figure S2). Four laboratories using Tianlong Nucleic Acid Extraction kit & BioGerm RT-PCR kits & Gentier PCR instruments were included, with the PCR efficiency ranging from 44.71 to 116.02% for ORF1ab and 87.80% to 169.15% for N, respectively (Table 4). Two laboratories using Tianlong Nucleic Acid Extraction kit & BioGerm RT-PCR kit & SLAN PCR instrument yielded efficiencies of 78.20% and 80.84% for ORF1ab and 98.62% and 100% for N (Table 4). For laboratories using Tianlong Nucleic Acid Extraction kit & BioGerm RT-PCR kit & ABI7500, the PCR efficiency varied with a minimum of 78.20% and a maximum of 217.47%, regardless of target genes (Table 4).

**Table 4:** PCR efficiencies of various laboratories using ORF1ab and N Ct values from EQA samples

Lab code	ORF1ab				N			
	1/slope	intercept	r^2^	*E* (%)	1/slope	intercept	r^2^	*E* (%)
Tianlong nucleic extraction kit & BioGerm RT-PCR kit & Gentier PCR instrument								
1110084	− 0.2827	43.86	0.9988	91.73	− 0.2737	45.56	0.9758	87.80
1110100	− 0.301	42.88	0.9796	99.99	− 0.301	44.72	1	99.99
1110155	− 0.1605	50.84	0.9995	44.71	− 0.301	43.64	1	99.99
110002B6	− 0.3345	42.39	0.9749	116.02	− 0.43	41.38	0.9423	169.15
Tianlong nucleic extraction kit & BioGerm RT-PCR kit & SLAN PCR instrument								
1110051	− 0.2573	44.1	0.993	80.84	− 0.3627	41.83	0.9862	130.52
1110091	− 0.2509	44.89	0.9908	78.20	− 0.3763	41.77	1	137.85
Tianlong nucleic extraction kit & BioGerm RT-PCR kit & ABI7500 PCR instrument								
1110006	− 0.301	42.05	0.9796	99.99	− 0.2676	44.71	0.9643	85.18
1110017	− 0.2737	43.36	0.9758	87.80	− 0.3345	43.57	0.9643	116.02
1110081	− 0.3763	40.04	0.9796	137.85	− 0.301	43.37	1	99.99
1110143	− 0.2573	43.99	0.9426	80.84	− 0.301	43.97	1	99.99
1110739	− 0.2509	44.89	0.9908	78.20	− 0.5017	40.65	0.9643	217.47

Lab, laboratory; r^2^, the coefficient of determination; E, efficiency

## Discussion

Reliable RT-PCR assays are essential for COVID-19 diagnosis [5, 25]. Many SARS-CoV-2 laboratories use open-system PCR-based methods established by different commercial extraction kits, RT-PCR kits, and PCR instruments [15]. The laboratories must confirm the test system's validity prior to use. The test quality can be continuously ensured by participating in EQA schemes [26]. In this study, we launched five EQA rounds between February 2022 and June 2022 in Beijing. The EQA samples contained concentrations near the limit of detection by the nucleic acid method. There was strong concordance among laboratories for the qualitative test result. Several EQA schemes for SARS-CoV-2 nucleic acid testing have been conducted nationally or regionally in China [14, 15, 27, 28]. The PPA and NPA were similar to the 2021 nationwide EQA for Delta variant [14] but higher than the 2020 nationwide EQA for non-variant SARS-CoV-2 [15]. It indicated that the testing capacity for SARS-CoV-2 was not impaired by omicron variant. The root cause analysis revealed that the incorrect qualitative interpretation of EQA results was mainly due to errors in data entry, mixed samples, and deficiencies in personnel operation. Besides, a few laboratories failed to detect the samples with low concentrations due to using analytically less sensitive methods. Thus, continual quality improvement is necessary [26].

The Ct values derived from SARS-CoV-2 RT-PCR have been associated with viral infectivity and used for isolation management [3, 9, 10, 13, 29–31]. Several parameters related to pre-analytic, analytic, and post-analytic phases affect the Ct values [18, 19, 24, 32, 33]. This study used EQA data to assess the comparability in Ct values derived from different test systems and laboratories. EQA data can avoid preanalytical issues and represents variations associated with RNA extraction, RT-PCR, and data analysis [33]. This study observed poor comparability in Ct values among different extraction kits, RT-PCR kits, and PCR instruments. These findings coincide with the published literature [18, 32, 34]. The variability in Ct values prevents direct comparability in Ct values among different test systems. This study observed a high likelihood of comparability in Ct values among laboratories using the same test system. These findings indicate that the variability of Ct value is more likely to be associated with diverse detection assays and less on their operation. To date, RT-PCR for SARS-CoV-2 authorized by the US Food and Drug Administration (FDA) and NMPA are interpreted qualitatively [35]. The qualitative test Ct values are not normalized to standardized controls of known concentration. Besides, multiple different SARS-CoV-2 target regions were detected simultaneously by certain tests, and each target may result in a different Ct value from the same specimen [36]. Additionally, there is a lack of international commutable quantitative reference standard material to harmonize assays across laboratories. As a result, Ct values generated by qualitative PCR tests should not be considered a quantitative measurement of viral load. The Ct values should not be used for clinical or epidemiological decision-making to avoid the potential for misinterpretation of the results [35–37]. Developing a quantitative SARS-CoV-2 RT-PCR assay that converts Ct values into copies/mL or IU/mL could overcome some limits [38].

Issues related to assay design, including primers, probe chemistry, enzymes, target selection, cycling conditions, and salt ion concentration, influence PCR efficiency [16, 39]. In support of this, a wide heterogeneity in PCR efficiency among different RT-PCR kits and inter-laboratories using the same test system was observed. Even though the difference in Ct values did not reach statistical significance among laboratories using the same test system, the PCR efficiency varied widely. This finding is quite different from that of Svec et al., who showed that the PCR efficiency was reproducibly stable on one platform [23]. The inter-laboratories variability of PCR efficiency can be ascribed to differences in technicians, batch effect, and variations among different laboratories.

There are flaws in this study. The dilution series used for the standard curves do not cover the upper range of measured quantities. Thus, the PCR efficiency calculated in this study may not reflect reality. Besides, only the most frequently used test systems were analyzed in this study when assessing variability in Ct values. A small number of laboratories were included for inter-laboratory comparison. Therefore, additional studies using extensive data may be necessary to validate the findings.

## Conclusions

In conclusion, there is strong concordance regarding the qualitative interpretation of RT-PCR assays for SARS-CoV-2 among different laboratories. A significant difference in Ct values was noted among different test systems. Ct values from qualitative RT-PCR tests should not be used for clinical or epidemiological decision-making to avoid the potential for misinterpretation of the results.

## Supplementary Information

**Additional file 1: Figure S1**. The standard curves were constructed using EQA samples detected by different RT-PCR kits within a laboratory.

**Additional file 2: Figure S2**. The standard curves were constructed using EQA samples detected by different laboratories.

**Additional file 3: Table S1**. The Ct values derived from EQA samples were detected by different RT-PCR kits. **Table S2**. The Ct values derived from EQA samples were detected by different extraction kits. **Table S3**. The Ct values derived from EQA samples were detected by different PCR instruments. **Table S4**. The Ct values derived from EQA samples were detected by different laboratories using the same test system.

## Data Availability

The data supporting the findings of this study are available from the corresponding author upon reasonable request.

## Abbreviations

RT-PCR: Real-time reverse transcriptase polymerase chain reaction
Ct: Cycle threshold
EQA: External quality assessment
SARS-CoV-2: Severe acute respiratory syndrome coronavirus 2
COVID-19: Coronavirus disease
VLPs: Virus-like particles
ANOVA: One-way analysis of variance
IQR: Interquartile range
SD: Standard deviation
PPA: Positive percentage agreement
NPA: Negative percentage agreement
