# Supplementary figures and images for: Assessing the comparability of cycle threshold values derived from five external quality assessment rounds for omicron nucleic acid testing
Source: Virol J. 2023 Jun 8;20:119. doi: 10.1186/s12985-023-02032-z (PMC10249569; doi:10.1186/s12985-023-02032-z)

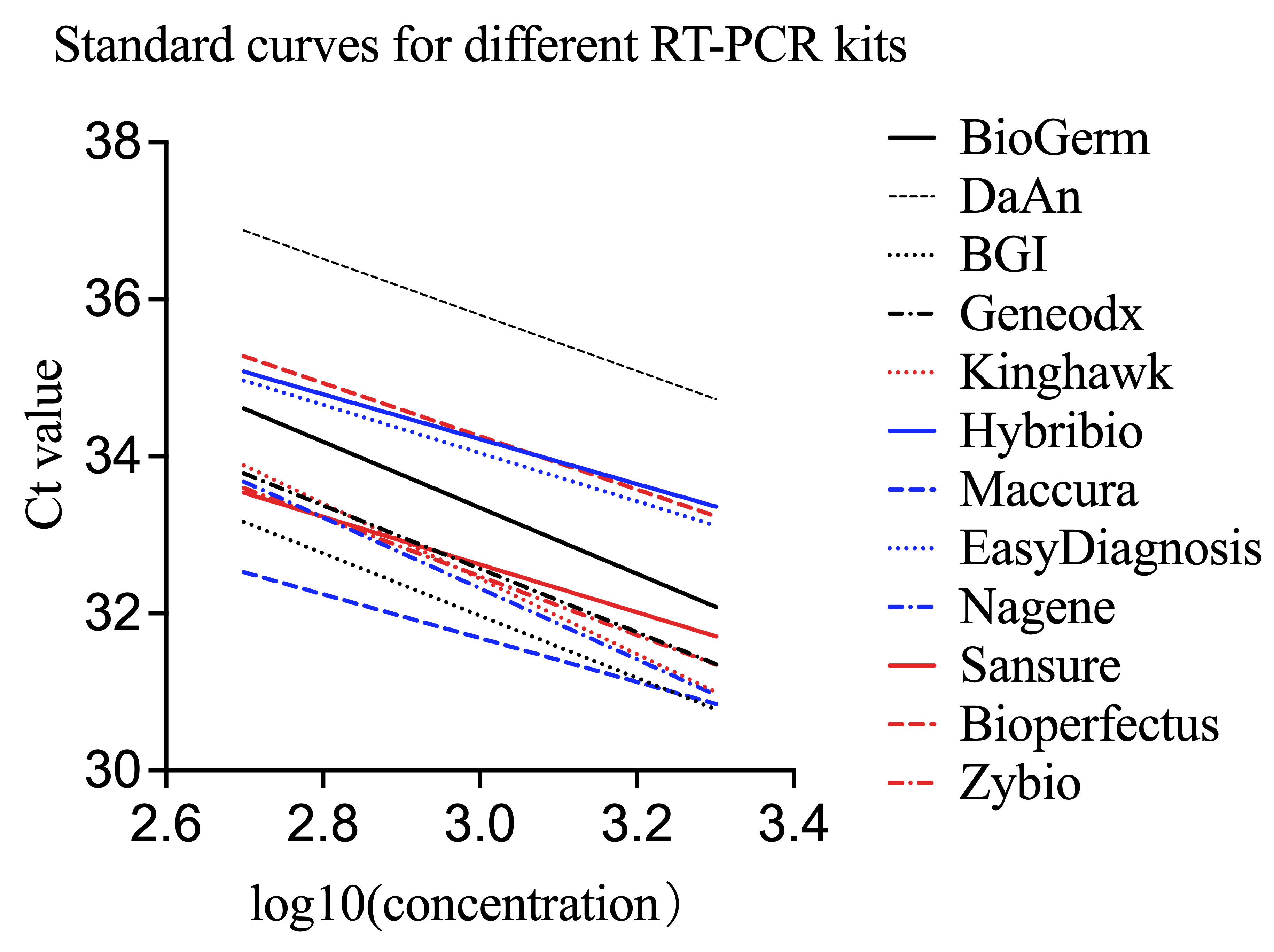

Supplement: Supplementary file 1 — Additional file 1: Figure S1. The standard curves were constructed using EQA samples detected by different RT-PCR kits within a laboratory. [file 12985_2023_2032_MOESM1_ESM.tiff]

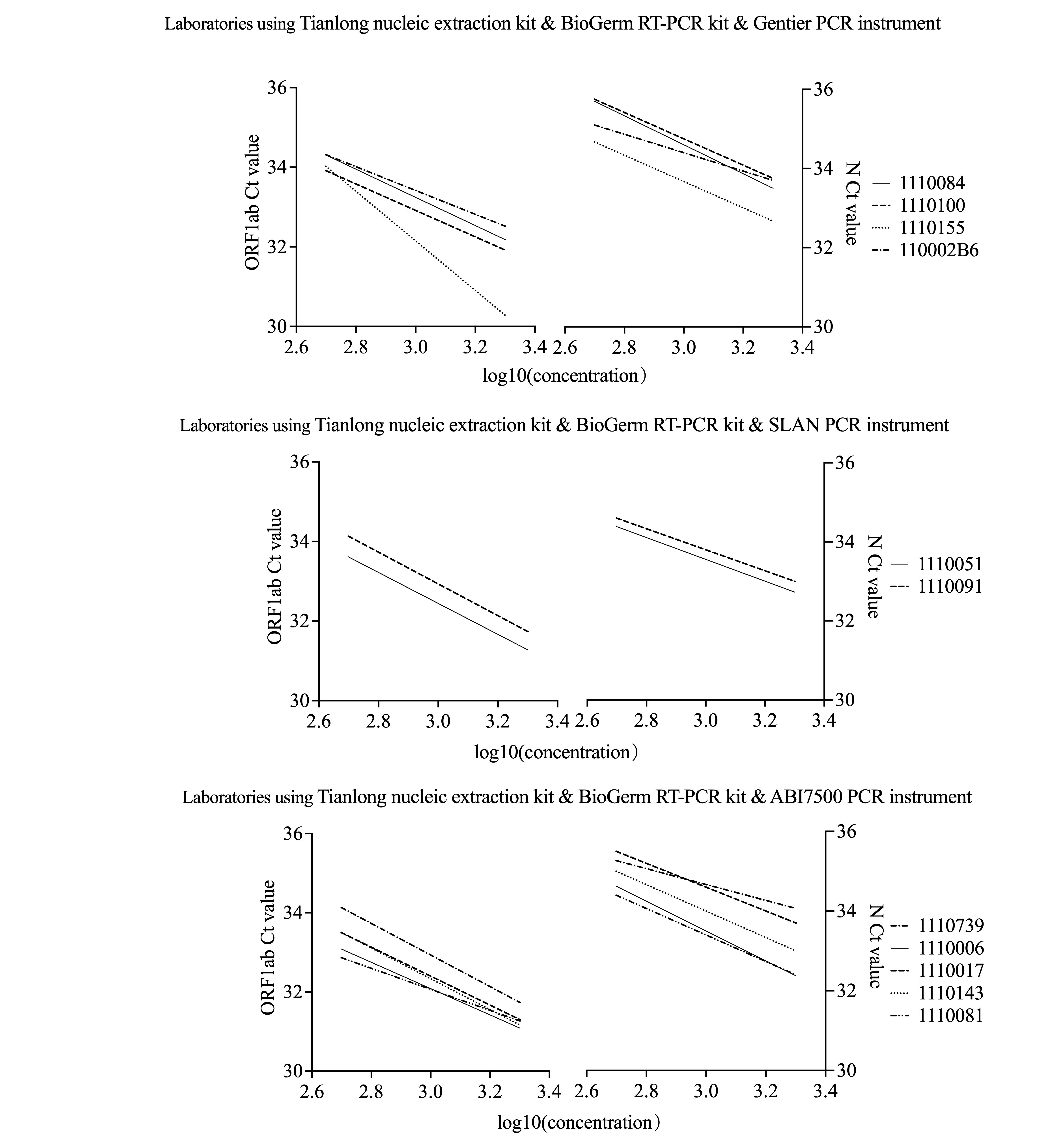

Supplement: Supplementary file 2 — Additional file 2: Figure S2. The standard curves were constructed using EQA samples detected by different laboratories. [file 12985_2023_2032_MOESM2_ESM.tiff]
