# Supplementary material for: Assessing the comparability of cycle threshold values derived from five external quality assessment rounds for omicron nucleic acid testing
Source: Virol J. 2023 Jun 8;20:119. doi: 10.1186/s12985-023-02032-z (PMC10249569; doi:10.1186/s12985-023-02032-z)
Supplement: Supplementary file 3 — Additional file 3: Table S1. The Ct values derived from EQA samples were detected by different RT-PCR kits. Table S2. The Ct values derived from EQA samples were detected by different extraction kits. Table S3. The Ct values derived from EQA samples were detected by different PCR instruments. Table S4. The Ct values derived from EQA samples were detected by different laboratories using the same test system. [file 12985_2023_2032_MOESM3_ESM.docx]

**Table S1. The Ct values derived from EQA samples were detected by different RT-PCR kits.**

| RT-PCR kits | 2.0×10^3^ copies/mL | | |  | 1.0×10^3^ copies/mL | | |  | 5.0×10^2^ copies/mL | | |  | 2.0×10^2^ copies/mL | | |
| --- | --- | --- | --- | --- | --- | --- | --- | --- | --- | --- | --- | --- | --- | --- | --- |
|  | mean | SD | Lab No. |  | mean | SD | Lab No. |  | mean | SD | Lab No. |  | mean | SD | Lab No. |
| Ct values for ORF1ab determined by different RT-PCR kits combined with different extraction kits and different PCR instruments | | | | | | | | | | | | | | | |
| BioGerm | 32.2 | 1.655 | 394 |  | 33.14 | 1.651 | 392 |  | 34.2 | 1.741 | 394 |  | 35.39 | 1.758 | 318 |
| DaAn | 33.41 | 2.085 | 119 |  | 34.5 | 2.004 | 119 |  | 35.46 | 2.102 | 119 |  | 36.63 | 1.963 | 100 |
| Kinghawk | 33.59 | 2.025 | 41 |  | 34.78 | 2.231 | 41 |  | 35 | 1.806 | 39 |  | 35.47 | 1.542 | 34 |
| Maccura | 30.77 | 1.166 | 13 |  | 31.77 | 1.092 | 13 |  | 32.62 | 1.261 | 13 |  | 34.27 | 1.191 | 11 |
| EasyDiagnosis | 32.38 | 2.003 | 63 |  | 33.22 | 2.549 | 63 |  | 34.21 | 1.752 | 63 |  | 35.45 | 1.572 | 47 |
| Sansure | 33.25 | 1.467 | 57 |  | 34.18 | 1.539 | 56 |  | 35.11 | 1.555 | 57 |  | 36.6 | 1.483 | 42 |
| Bioperfectus | 32.84 | 1.89 | 83 |  | 33.9 | 1.785 | 83 |  | 34.88 | 1.978 | 83 |  | 35.77 | 1.569 | 65 |
| Zybio | 30.91 | 2.454 | 67 |  | 31.73 | 2.403 | 67 |  | 32.72 | 2.295 | 67 |  | 33.61 | 2.474 | 51 |
| ABT | 32 | 2.194 | 97 |  | 33 | 2.005 | 97 |  | 33.86 | 2.116 | 97 |  | 34.94 | 2.108 | 79 |
|  |  |  |  |  |  |  |  |  |  |  |  |  |  |  |  |
| Ct values for N determined by different RT-PCR kits combined with different extraction kits and different PCR instruments | | | | | | | | | | | | | | | |
| BioGerm | 33.13 | 1.632 | 394 |  | 34.01 | 1.608 | 393 |  | 34.8 | 1.619 | 394 |  | 35.79 | 1.59 | 318 |
| DaAn | 34.81 | 2.293 | 119 |  | 35.75 | 2.128 | 119 |  | 36.48 | 2.107 | 119 |  | 37.42 | 1.602 | 95 |
| Kinghawk | 33.15 | 1.931 | 41 |  | 34.02 | 1.796 | 41 |  | 34.9 | 1.786 | 41 |  | 35.74 | 1.238 | 34 |
| Maccura | 32.23 | 1.235 | 13 |  | 33 | 1.291 | 13 |  | 34 | 1.225 | 13 |  | 35.55 | 0.8202 | 11 |
| EasyDiagnosis | 33.6 | 2.004 | 63 |  | 34.06 | 2.925 | 62 |  | 35.05 | 2.128 | 63 |  | 36.2 | 1.673 | 45 |
| Sansure | 31.46 | 1.712 | 57 |  | 32.43 | 1.746 | 56 |  | 33.33 | 2.174 | 57 |  | 34.71 | 1.47 | 42 |
| Bioperfectus | 33.94 | 2.329 | 83 |  | 35.05 | 2.23 | 83 |  | 35.81 | 2.033 | 83 |  | 36.83 | 1.79 | 65 |
| Zybio | 31.34 | 2.403 | 67 |  | 32.28 | 2.275 | 67 |  | 33.24 | 2.29 | 67 |  | 34.14 | 2.506 | 51 |
| ABT | 32 | 2.184 | 97 |  | 32.85 | 2.547 | 97 |  | 33.88 | 2.157 | 97 |  | 35.15 | 1.987 | 78 |
|  |  |  |  |  |  |  |  |  |  |  |  |  |  |  |  |
| Ct values for ORF1ab determined by different RT-PCR kits combined with the Tianlong nucleic extraction kit and different PCR instruments | | | | | | | | | | | | | | | |
| BioGerm | 31.86 | 1.388 | 112 |  | 32.81 | 1.366 | 112 |  | 33.85 | 1.409 | 113 |  | 35.12 | 1.577 | 95 |
| DaAn | 33.05 | 1.431 | 21 |  | 34.24 | 1.3 | 21 |  | 35.43 | 1.434 | 21 |  | 36.75 | 1.528 | 16 |
| Kinghawk | 33.56 | 2.093 | 18 |  | 34.61 | 2.38 | 18 |  | 35.11 | 1.811 | 18 |  | 35.47 | 1.457 | 15 |
| Maccura | 30.2 | 1.304 | 5 |  | 31.4 | 0.894 | 5 |  | 32.2 | 1.304 | 5 |  | 33.75 | 0.5 | 4 |
| EasyDiagnosis | 31.6 | 1.517 | 5 |  | 32.6 | 0.894 | 5 |  | 34 | 1.225 | 5 |  | 35 | 1.414 | 4 |
| Zybio | 30.38 | 1.996 | 8 |  | 31.63 | 1.408 | 8 |  | 32.25 | 2.55 | 8 |  | 33.17 | 2.317 | 6 |
| ABT | 31.73 | 2.187 | 15 |  | 32.73 | 1.87 | 15 |  | 33.33 | 1.589 | 15 |  | 34 | 1.206 | 12 |
|  |  |  |  |  |  |  |  |  |  |  |  |  |  |  |  |
| Ct values for N determined by different RT-PCR kits combined with the Tianlong nucleic extraction kit and different PCR instruments | | | | | | | | | | | | | | | |
| BioGerm | 33.01 | 1.597 | 112 |  | 33.89 | 1.516 | 111 |  | 34.69 | 1.421 | 112 |  | 35.69 | 1.452 | 94 |
| DaAn | 35.19 | 1.537 | 21 |  | 36.24 | 1.338 | 21 |  | 36.71 | 1.488 | 21 |  | 37.38 | 1.36 | 16 |
| Kinghawk | 33.11 | 2.246 | 18 |  | 33.94 | 2.014 | 18 |  | 34.61 | 1.65 | 18 |  | 35.6 | 1.242 | 15 |
| Maccura | 31.6 | 0.8944 | 5 |  | 32.8 | 1.304 | 5 |  | 33.8 | 1.304 | 5 |  | 35.25 | 0.9574 | 4 |
| EasyDiagnosis | 34 | 1.414 | 5 |  | 35 | 1.225 | 5 |  | 35.4 | 0.8944 | 5 |  | 36.33 | 1.155 | 3 |
| Zybio | 30.38 | 3.068 | 8 |  | 31.63 | 3.159 | 8 |  | 32.25 | 3.536 | 8 |  | 33 | 3.521 | 6 |
| ABT | 31.67 | 2.257 | 15 |  | 32.8 | 1.897 | 15 |  | 33.67 | 1.633 | 15 |  | 34.5 | 1.243 | 12 |
|  |  |  |  |  |  |  |  |  |  |  |  |  |  |  |  |
| Ct values for ORF1ab determined by different RT-PCR kits combined with Tianlong nucleic extraction kit and ABI7500 PCR instrument | | | | | | | | | | | | | | | |
| BioGerm | 31.79 | 1.673 | 33 |  | 32.79 | 1.556 | 33 |  | 33.76 | 1.601 | 33 |  | 35.26 | 1.607 | 27 |
| DaAn | 33.38 | 1.506 | 8 |  | 34.63 | 1.188 | 8 |  | 35.88 | 1.553 | 8 |  | 37.33 | 1.033 | 6 |
| Kinghawk | 32.6 | 2.966 | 5 |  | 34 | 3.317 | 5 |  | 34 | 1.581 | 5 |  | 34.75 | 2.062 | 4 |
| Maccura | 30.2 | 1.304 | 5 |  | 31.4 | 0.8944 | 5 |  | 32.2 | 1.304 | 5 |  | 33.75 | 0.5 | 4 |
| ABT | 32.5 | 3.271 | 6 |  | 33.17 | 2.639 | 6 |  | 33.67 | 2.251 | 6 |  | 34.25 | 1.893 | 4 |
|  |  |  |  |  |  |  |  |  |  |  |  |  |  |  |  |
| Ct values for N determined by different RT-PCR kits combined with Tianlong nucleic extraction kit and ABI7500 PCR instrument | | | | | | | | | | | | | | | |
| BioGerm | 33.27 | 1.485 | 33 |  | 34.24 | 1.324 | 33 |  | 34.88 | 1.219 | 33 |  | 36.04 | 1.315 | 27 |
| DaAn | 34.5 | 1.069 | 8 |  | 35.63 | 1.061 | 8 |  | 36.13 | 1.356 | 8 |  | 37 | 1.095 | 6 |
| Kinghawk | 32.4 | 1.342 | 5 |  | 33.4 | 1.342 | 5 |  | 34.6 | 1.517 | 5 |  | 35 | 1.155 | 4 |
| Maccura | 31.6 | 0.8944 | 5 |  | 32.8 | 1.304 | 5 |  | 33.8 | 1.304 | 5 |  | 35.25 | 0.9574 | 4 |
| ABT | 31.17 | 2.137 | 6 |  | 32.5 | 2.074 | 6 |  | 33.33 | 1.506 | 6 |  | 33.75 | 0.9574 | 4 |
|  |  |  |  |  |  |  |  |  |  |  |  |  |  |  |  |
| Ct values for ORF1ab determined by different RT-PCR kits combined with the DaAn nucleic extraction kit and different PCR instruments | | | | | | | | | | | | | | | |
| BioGerm | 32.73 | 0.6467 | 11 |  | 34.27 | 1.489 | 11 |  | 35.73 | 1.421 | 11 |  | 37.14 | 1.864 | 7 |
| DaAn | 33.47 | 2.048 | 32 |  | 34.47 | 2.17 | 32 |  | 35.34 | 1.928 | 32 |  | 36.19 | 2.6 | 31 |
| EasyDiagnosis | 33.33 | 1.033 | 6 |  | 34.17 | 1.472 | 6 |  | 35.17 | 0.983 | 6 |  | 36.75 | 1.258 | 4 |
| Sansure | 33.14 | 2.61 | 7 |  | 33.33 | 2.944 | 6 |  | 35 | 2.708 | 7 |  | 38 | 1.871 | 5 |
| ABT | 32.5 | 2.221 | 16 |  | 33.5 | 2 | 16 |  | 34.25 | 2.113 | 16 |  | 35.08 | 1.656 | 13 |
|  |  |  |  |  |  |  |  |  |  |  |  |  |  |  |  |
| Ct values for N determined by different RT-PCR kits combined with the DaAn nucleic extraction kit and different PCR instruments | | | | | | | | | | | | | | | |
| BioGerm | 33.45 | 1.214 | 11 |  | 34.82 | 1.079 | 11 |  | 35.45 | 1.128 | 11 |  | 36.14 | 1.215 | 7 |
| DaAn | 34.69 | 2.086 | 32 |  | 35.66 | 1.825 | 32 |  | 36.47 | 1.704 | 32 |  | 37.69 | 1.289 | 26 |
| EasyDiagnosis | 34.83 | 1.472 | 6 |  | 33.5 | 4.231 | 6 |  | 36.17 | 1.722 | 6 |  | 37.33 | 1.155 | 3 |
| Sansure | 31 | 2.449 | 7 |  | 31.33 | 2.944 | 6 |  | 33 | 2.769 | 7 |  | 35.6 | 1.342 | 5 |
| ABT | 32 | 1.897 | 16 |  | 33 | 1.966 | 16 |  | 34.13 | 1.928 | 16 |  | 35.15 | 1.819 | 13 |

Abbreviations: SD, standard deviation; No., number; Ct, cycle threshold.

**Table S2. The Ct values derived from EQA samples were detected by different extraction kits.**

| Extraction kits | 2.0×10^3^ copies/mL | | |  | 1.0×10^3^ copies/mL | | |  | 5.0×10^2^ copies/mL | | |  | 2.0×10^2^ copies/mL | | |
| --- | --- | --- | --- | --- | --- | --- | --- | --- | --- | --- | --- | --- | --- | --- | --- |
|  | mean | SD | Lab No. |  | mean | SD | Lab No. |  | mean | SD | Lab No. |  | mean | SD | Lab No. |
| Ct values for ORF1ab determined by different extraction kits combined with BioGerm RT-PCR kits and different PCR instruments | | | | | | | | | | | | | | | |
| BioTeke | 32.6 | 1.174 | 10 |  | 33.7 | 0.8233 | 10 |  | 34.8 | 1.135 | 10 |  | 36.25 | 1.669 | 8 |
| BioGerm | 33.11 | 1.696 | 37 |  | 34.05 | 1.433 | 37 |  | 34.78 | 1.601 | 37 |  | 36.45 | 1.549 | 29 |
| bioer | 31.88 | 1.5 | 16 |  | 32.81 | 1.377 | 16 |  | 34 | 1.033 | 16 |  | 34.92 | 1.115 | 13 |
| DaAn | 32.73 | 0.6467 | 11 |  | 34.27 | 1.489 | 11 |  | 35.73 | 1.421 | 11 |  | 37.14 | 1.864 | 7 |
| genfine | 33.11 | 1.833 | 9 |  | 33.44 | 1.59 | 9 |  | 34.33 | 2.291 | 9 |  | 36.57 | 1.902 | 7 |
| genmabio | 32.56 | 1.153 | 16 |  | 33.75 | 1 | 16 |  | 35 | 1.414 | 16 |  | 35.71 | 0.995 | 14 |
| ComWin | 33 | 2.582 | 10 |  | 33.44 | 1.59 | 9 |  | 34.4 | 1.838 | 10 |  | 35.38 | 1.598 | 8 |
| Maccura | 34 | 1 | 3 |  | 35.67 | 1.155 | 3 |  | 36.33 | 1.155 | 3 |  | 36.67 | 1.155 | 3 |
| NanoMagBio | 31.2 | 0.8367 | 5 |  | 32.2 | 0.8367 | 5 |  | 33.8 | 0.8367 | 5 |  | 35 | 0 | 4 |
| Sansure | 32 | 1.414 | 4 |  | 32.75 | 1.893 | 4 |  | 34.25 | 2.217 | 4 |  | 36 | 1.732 | 3 |
| Bioperfectus | 31.71 | 1.47 | 42 |  | 32.55 | 1.783 | 42 |  | 33.62 | 2.024 | 42 |  | 34.68 | 2.128 | 34 |
| Tianlong | 31.86 | 1.388 | 112 |  | 32.81 | 1.366 | 112 |  | 33.85 | 1.409 | 113 |  | 35.12 | 1.577 | 95 |
| geneonbio | 30 | 1.826 | 4 |  | 30.75 | 1.5 | 4 |  | 32 | 1.155 | 4 |  | 33 | 1.732 | 3 |
| ZhongkeBio | 32.4 | 0.9947 | 20 |  | 33.3 | 0.9787 | 20 |  | 34.65 | 0.8751 | 20 |  | 35.44 | 0.727 | 16 |
| Zybio | 31.73 | 2.119 | 49 |  | 32.71 | 2.17 | 49 |  | 33.9 | 2.33 | 49 |  | 35.05 | 2.109 | 41 |
|  |  |  |  |  |  |  |  |  |  |  |  |  |  |  |  |
| Ct values for N determined by different extraction kits combined with BioGerm RT-PCR kits and different PCR instruments | | | | | | | | | | | | | | | |
| BioTeke | 31.88 | 1.5 | 10 |  | 34.4 | 1.075 | 10 |  | 35.3 | 1.16 | 10 |  | 36.38 | 1.408 | 8 |
| BioGerm | 33.45 | 1.214 | 37 |  | 34.49 | 1.325 | 37 |  | 35.3 | 1.488 | 37 |  | 36.41 | 1.268 | 29 |
| bioer | 33 | 1 | 16 |  | 34.13 | 1.204 | 16 |  | 35.13 | 1.408 | 16 |  | 35.77 | 1.363 | 13 |
| DaAn | 32.78 | 3.492 | 11 |  | 34.82 | 1.079 | 11 |  | 35.45 | 1.128 | 11 |  | 36.14 | 1.215 | 7 |
| genfine | 33.8 | 2.098 | 9 |  | 33.56 | 2.651 | 9 |  | 34.33 | 2.915 | 9 |  | 35.57 | 2.225 | 7 |
| genmabio | 34 | 1 | 16 |  | 34.88 | 1.544 | 16 |  | 35.75 | 1.77 | 16 |  | 36.43 | 1.342 | 14 |
| ComWin | 32.6 | 0.8944 | 10 |  | 34.5 | 1.9 | 10 |  | 35.3 | 2.003 | 10 |  | 36.25 | 1.669 | 8 |
| Maccura | 33 | 0.8165 | 3 |  | 35 | 1 | 3 |  | 36 | 0 | 3 |  | 36.33 | 1.155 | 3 |
| NanoMagBio | 32.62 | 2.06 | 5 |  | 33.8 | 1.304 | 5 |  | 34.2 | 0.8367 | 5 |  | 35.75 | 1.258 | 4 |
| Sansure | 33.01 | 1.597 | 4 |  | 34 | 0.8165 | 4 |  | 34.75 | 0.9574 | 4 |  | 35.33 | 0.5774 | 3 |
| Bioperfectus | 33.53 | 1.124 | 42 |  | 33.43 | 2.221 | 42 |  | 34.19 | 2.255 | 42 |  | 35.06 | 2.449 | 34 |
| Tianlong | 31.75 | 1.5 | 112 |  | 33.89 | 1.516 | 111 |  | 34.69 | 1.421 | 112 |  | 35.69 | 1.452 | 94 |
| geneonbio | 32.78 | 1.531 | 4 |  | 33 | 1.826 | 4 |  | 33.75 | 1.5 | 4 |  | 34.33 | 1.528 | 3 |
| ZhongkeBio | 32.85 | 1.182 | 20 |  | 33.6 | 0.9947 | 20 |  | 34.55 | 1.146 | 20 |  | 35.38 | 1.455 | 16 |
| Zybio | 32.78 | 1.531 | 49 |  | 33.76 | 1.762 | 49 |  | 34.57 | 1.744 | 49 |  | 35.61 | 1.481 | 41 |
| Zhijiang | 32.85 | 1.182 | 19 |  | 34.32 | 0.8852 | 19 |  | 34.89 | 0.8753 | 19 |  | 36 | 0.9535 | 12 |
|  |  |  |  |  |  |  |  |  |  |  |  |  |  |  |  |
| Ct values for ORF1ab determined by different extraction kits combined with BioGerm RT-PCR kits and ABI7500 PCR instrument | | | | | | | | | | | | | | | |
| BioGerm | 33.29 | 1.707 | 21 |  | 34.14 | 1.59 | 21 |  | 34.9 | 1.868 | 21 |  | 36.65 | 1.835 | 17 |
| bioer | 31.67 | 1.323 | 9 |  | 32.67 | 1 | 9 |  | 34.11 | 0.7817 | 9 |  | 35.14 | 0.8997 | 7 |
| genfine | 32 | 1.633 | 4 |  | 32.5 | 1.915 | 4 |  | 33.25 | 2.986 | 4 |  | 36.67 | 2.887 | 3 |
| genmabio | 32.86 | 1.345 | 7 |  | 34 | 1.291 | 7 |  | 35.29 | 1.604 | 7 |  | 36.14 | 1.069 | 7 |
| lepu | 32 | 2 | 3 |  | 32.33 | 2.082 | 3 |  | 33.67 | 2.082 | 3 |  | 36 | 2 | 3 |
| Bioperfectus | 32.7 | 1.767 | 10 |  | 33.5 | 1.958 | 10 |  | 35 | 1.944 | 10 |  | 35.25 | 1.488 | 8 |
| Tianlong | 31.79 | 1.673 | 33 |  | 32.79 | 1.556 | 33 |  | 33.76 | 1.601 | 33 |  | 35.26 | 1.607 | 27 |
| Zhijiang | 32.75 | 1.258 | 4 |  | 33.25 | 0.9574 | 4 |  | 34.25 | 0.9574 | 4 |  | 35.33 | 0.5774 | 3 |
| ZhongkeBio | 32.71 | 0.7263 | 14 |  | 33.57 | 0.8516 | 14 |  | 34.71 | 0.7263 | 14 |  | 35.64 | 0.6742 | 11 |
| Zybio | 31.54 | 2.47 | 13 |  | 32.69 | 2.562 | 13 |  | 34.31 | 2.25 | 13 |  | 35.36 | 2.873 | 11 |
|  |  |  |  |  |  |  |  |  |  |  |  |  |  |  |  |
| Ct values for N determined by different extraction kits combined with BioGerm RT-PCR kits and ABI7500 PCR instrument | | | | | | | | | | | | | | | |
| BioGerm | 34.05 | 1.564 | 21 |  | 34.52 | 1.601 | 21 |  | 35.24 | 1.841 | 21 |  | 36.29 | 1.49 | 17 |
| bioer | 33.56 | 1.424 | 9 |  | 34.33 | 1.118 | 9 |  | 35.67 | 1.118 | 9 |  | 36.14 | 0.8997 | 7 |
| genfine | 30.5 | 4.123 | 4 |  | 31.75 | 3.202 | 4 |  | 32.5 | 3.697 | 4 |  | 35 | 3.606 | 3 |
| genmabio | 34.43 | 1.397 | 7 |  | 35.57 | 1.512 | 7 |  | 36.29 | 1.704 | 7 |  | 36.71 | 1.38 | 7 |
| lepu | 33 | 1 | 3 |  | 34.33 | 2.082 | 3 |  | 34.67 | 2.082 | 3 |  | 36 | 1.732 | 3 |
| Bioperfectus | 32.9 | 2.234 | 10 |  | 33.9 | 2.424 | 10 |  | 34.8 | 2.348 | 10 |  | 36.13 | 1.246 | 8 |
| Tianlong | 33.27 | 1.485 | 33 |  | 34.24 | 1.324 | 33 |  | 34.88 | 1.219 | 33 |  | 36.04 | 1.315 | 27 |
| Zhijiang | 33.5 | 0.5774 | 4 |  | 34.5 | 0.5774 | 4 |  | 35 | 1.155 | 4 |  | 36.67 | 0.5774 | 3 |
| ZhongkeBio | 33.14 | 1.231 | 14 |  | 33.86 | 1.027 | 14 |  | 34.64 | 1.277 | 14 |  | 35.64 | 1.502 | 11 |
| Zybio | 32.38 | 1.502 | 13 |  | 33.69 | 1.843 | 13 |  | 34.38 | 1.758 | 13 |  | 35 | 1.549 | 11 |
|  |  |  |  |  |  |  |  |  |  |  |  |  |  |  |  |
| Ct values for ORF1ab determined by different extraction kits combined with BioGerm RT-PCR kits and SLAN PCR instrument | | | | | | | | | | | | | | | |
| BioGerm | 33.11 | 0.6009 | 9 |  | 34.44 | 0.8819 | 9 |  | 35.11 | 0.928 | 9 |  | 36.67 | 0.5164 | 6 |
| Bioperfectus | 32.17 | 1.329 | 6 |  | 33 | 1.095 | 6 |  | 33.5 | 0.8367 | 6 |  | 35 | 0.8165 | 4 |
| Tianlong | 32.13 | 1.204 | 31 |  | 33.06 | 1.413 | 31 |  | 34.06 | 1.162 | 32 |  | 35.43 | 1.55 | 28 |
| Zhijiang | 32.8 | 0.8367 | 5 |  | 34.2 | 0.4472 | 5 |  | 34 | 0.7071 | 5 |  | 35.33 | 1.155 | 3 |
| Zybio | 32.4 | 0.8433 | 10 |  | 33.1 | 0.7379 | 10 |  | 34.5 | 0.8498 | 10 |  | 35.56 | 0.7265 | 9 |
| BioTeke | 32.8 | 1.304 | 5 |  | 33.6 | 0.8944 | 5 |  | 34.8 | 1.483 | 5 |  | 36.25 | 1.708 | 4 |
| DaAn | 32.7 | 0.6749 | 10 |  | 34.2 | 1.549 | 10 |  | 35.6 | 1.43 | 10 |  | 37.14 | 1.864 | 7 |
| ComWin | 33.2 | 3.701 | 5 |  | 32.75 | 1.708 | 4 |  | 34.4 | 2.702 | 5 |  | 35.25 | 2.062 | 4 |
|  |  |  |  |  |  |  |  |  |  |  |  |  |  |  |  |
| Ct values for N determined by different extraction kits combined with BioGerm RT-PCR kits and SLAN PCR instrument | | | | | | | | | | | | | | | |
| BioGerm | 33.67 | 0.7071 | 9 |  | 34.56 | 0.527 | 9 |  | 35.44 | 0.527 | 9 |  | 36.33 | 1.033 | 6 |
| Bioperfectus | 33.17 | 0.7528 | 6 |  | 34.5 | 0.8367 | 6 |  | 34.83 | 0.7528 | 6 |  | 36.25 | 0.9574 | 4 |
| Tianlong | 33.1 | 1.274 | 31 |  | 34 | 1.285 | 24 |  | 34.74 | 1.154 | 31 |  | 35.7 | 1.137 | 27 |
| Zhijiang | 34 | 1 | 5 |  | 34.6 | 0.8944 | 5 |  | 35 | 0.7071 | 5 |  | 35.67 | 1.155 | 3 |
| Zybio | 33.3 | 0.9487 | 10 |  | 34.5 | 1.08 | 10 |  | 35.4 | 1.075 | 10 |  | 36.33 | 0.7071 | 9 |
| BioTeke | 33.8 | 1.304 | 5 |  | 34.8 | 1.095 | 5 |  | 35.2 | 0.8367 | 5 |  | 36.5 | 1 | 4 |
| DaAn | 33.3 | 1.16 | 10 |  | 34.2 | 1.095 | 5 |  | 35.3 | 1.059 | 10 |  | 36.14 | 1.215 | 7 |
| ComWin | 33.4 | 2.881 | 5 |  | 34 | 2.449 | 5 |  | 34.6 | 2.702 | 5 |  | 35.5 | 2.082 | 4 |

Abbreviations: SD, standard deviation; No., number; Ct, cycle threshold; Lab, laboratory.

**Table S3. The Ct values derived from EQA samples were detected by different PCR instruments.**

| PCR instruments | 2.0×10^3^ copies/mL | | |  | 1.0×10^3^ copies/mL | | |  | 5.0×10^2^ copies/mL | | |  | 2.0×10^2^ copies/mL | | |
| --- | --- | --- | --- | --- | --- | --- | --- | --- | --- | --- | --- | --- | --- | --- | --- |
|  | mean | SD | Lab No. |  | mean | SD | Lab No. |  | mean | SD | Lab No. |  | mean | SD | Lab No. |
| Ct values for ORF1ab determined by Tianlong nucleic extraction kit and BioGerm RT-PCR kit combined with different PCR instruments | | | | | | | | | | | | | | | |
| ABI 7500 | 31.79 | 1.673 | 33 |  | 32.79 | 1.556 | 33 |  | 33.76 | 1.601 | 33 |  | 35.26 | 1.607 | 27 |
| Roche Light Cycler 480 | 31.13 | 1.808 | 8 |  | 31.5 | 1.195 | 8 |  | 32.5 | 1.309 | 8 |  | 33 | 1.155 | 7 |
| SLAN | 32.13 | 1.204 | 31 |  | 33.06 | 1.413 | 31 |  | 34.06 | 1.162 | 32 |  | 35.43 | 1.55 | 28 |
| Gentier | 31.85 | 1.149 | 33 |  | 32.85 | 1.093 | 33 |  | 33.97 | 1.334 | 33 |  | 35.15 | 1.379 | 27 |
|  |  |  |  |  |  |  |  |  |  |  |  |  |  |  |  |
| Ct values for N determined by Tianlong nucleic extraction kit and BioGerm RT-PCR kit combined with different PCR instruments | | | | | | | | | | | | | | | |
| ABI 7500 | 33.27 | 1.485 | 33 |  | 34.24 | 1.324 | 33 |  | 34.88 | 1.219 | 33 |  | 36.04 | 1.315 | 27 |
| Roche Light Cycler 480 | 31.75 | 1.982 | 8 |  | 32.38 | 1.188 | 8 |  | 33.38 | 0.744 | 8 |  | 34.29 | 0.488 | 7 |
| SLAN | 33.1 | 1.274 | 31 |  | 33.93 | 1.258 | 30 |  | 34.74 | 1.154 | 31 |  | 35.7 | 1.137 | 27 |
| Gentier | 32.94 | 1.87 | 33 |  | 33.88 | 1.867 | 33 |  | 34.67 | 1.831 | 33 |  | 35.67 | 1.881 | 27 |
|  |  |  |  |  |  |  |  |  |  |  |  |  |  |  |  |
| Ct values for ORF1ab determined by Zybio nucleic acid extraction kit and BioGerm RT-PCR kit combined with different PCR instruments | | | | | | | | | | | | | | | |
| ABI 7500 | 31.54 | 2.47 | 13 |  | 32.69 | 2.562 | 13 |  | 34.31 | 2.25 | 13 |  | 35.36 | 2.873 | 11 |
| Roche Light Cycler 480 | 30.33 | 2.958 | 9 |  | 31.22 | 2.906 | 9 |  | 31.56 | 3.127 | 9 |  | 34.43 | 2.37 | 7 |
| SLAN | 32.4 | 0.8433 | 10 |  | 33.1 | 0.7379 | 10 |  | 34.5 | 0.8498 | 10 |  | 35.56 | 0.7265 | 9 |
| Bio-Rad CFX96 | 33 | 2 | 5 |  | 33.8 | 2.49 | 5 |  | 35.6 | 2.302 | 5 |  | 35.25 | 2.217 | 4 |
|  |  |  |  |  |  |  |  |  |  |  |  |  |  |  |  |
| Ct values for N determined by Zybio nucleic acid extraction kit and BioGerm RT-PCR kit combined with different PCR instruments | | | | | | | | | | | | | | | |
| ABI 7500 | 32.38 | 1.502 | 13 |  | 33.69 | 1.843 | 13 |  | 34.38 | 1.758 | 13 |  | 35 | 1.549 | 11 |
| Roche Light Cycler 480 | 31.78 | 1.986 | 9 |  | 32.44 | 2.404 | 9 |  | 33 | 1.936 | 9 |  | 34.86 | 1.574 | 7 |
| SLAN | 33.3 | 0.9487 | 10 |  | 34.5 | 1.08 | 10 |  | 35.4 | 1.075 | 10 |  | 36.33 | 0.7071 | 9 |
| Bio-Rad CFX96 | 34.4 | 0.8944 | 5 |  | 35 | 1.225 | 5 |  | 36.2 | 1.304 | 5 |  | 37.5 | 1.291 | 4 |

Abbreviations: SD, standard deviation; No., number; Ct, cycle threshold; Lab, laboratory.

**Table S4. The Ct values derived from EQA samples were detected by different laboratories using the same test system.**

| Lab code | 2.0×10^3^ copies/mL | | |  | 1.0×10^3^ copies/mL | | |  | 5.0×10^2^ copies/mL | | |  | 2.0×10^2^ copies/mL | | |
| --- | --- | --- | --- | --- | --- | --- | --- | --- | --- | --- | --- | --- | --- | --- | --- |
|  | mean | SD | sample No. |  | mean | SD | sample No. |  | mean | SD | sample No. |  | mean | SD | sample No. |
| Ct values for ORF1ab determined by Tianlong nucleic extraction kit and BioGerm RT-PCR kit combined with Gentier PCR instrument | | | | | | | | | | | | | | | |
| 1110084 | 32.2 | 0.8367 | 5 |  | 33.2 | 0.8367 | 5 |  | 34.33 | 1.528 | 5 |  | 36 | 0.8165 | 4 |
| 1110100 | 32 | 0.8165 | 4 |  | 32.75 | 0.5 | 4 |  | 34 | 0 | 4 |  | 35 | 0 | 3 |
| 1110155 | 30.25 | 1.708 | 4 |  | 32.2 | 1.304 | 5 |  | 34 | 0.8165 | 4 |  | 33.75 | 2.63 | 4 |
| 1111187 | 32.6 | 0.8944 | 5 |  | 33.6 | 1.673 | 5 |  | 32.25 | 2.217 | 5 |  | 35.5 | 1.291 | 4 |
| 110002B6 | 32.6 | 0.5477 | 5 |  | 33.25 | 0.5 | 4 |  | 34.4 | 1.817 | 5 |  | 35.75 | 0.9574 | 4 |
| 110046B1 | 31.33 | 0.5774 | 3 |  | 32 | 1 | 3 |  | 34.4 | 0.5477 | 3 |  | 34.67 | 1.155 | 3 |
|  |  |  |  |  |  |  |  |  |  |  |  |  |  |  |  |
| Ct values for ORF1ab determined by Tianlong nucleic extraction kit and BioGerm RT-PCR kit combined with SLAN PCR instrument | | | | | | | | | | | | | | | |
| 1110051 | 31.33 | 0.5774 | 3 |  | 32.33 | 0.5774 | 3 |  | 33.67 | 0.5774 | 3 |  | 34 | 1 | 3 |
| 1110057 | 32.4 | 0.8944 | 5 |  | 34 | 1.633 | 4 |  | 34.4 | 1.342 | 5 |  | 35.25 | 0.5 | 4 |
| 1110091 | 31.8 | 0.8367 | 5 |  | 32.8 | 1.643 | 5 |  | 34.2 | 1.095 | 5 |  | 36 | 1.826 | 4 |
|  |  |  |  |  |  |  |  |  |  |  |  |  |  |  |  |
| Ct values for ORF1ab determined by Tianlong nucleic extraction kit and BioGerm RT-PCR kit combined with ABI 7500 PCR instrument | | | | | | | | | | | | | | | |
| 1110006 | 31 | 1.155 | 4 |  | 32.25 | 0.9574 | 4 |  | 33 | 0.8165 | 4 |  | 34.67 | 1.155 | 3 |
| 1110011 | 32.33 | 1.155 | 3 |  | 33.33 | 1.528 | 3 |  | 34.33 | 0.5774 | 3 |  | 36.33 | 1.528 | 3 |
| 1110017 | 31.2 | 0.4472 | 5 |  | 32.6 | 1.14 | 5 |  | 33.4 | 0.5477 | 5 |  | 35.25 | 0.9574 | 4 |
| 1110081 | 31.2 | 1.304 | 5 |  | 32.2 | 1.304 | 5 |  | 32.8 | 2.168 | 5 |  | 34.5 | 1 | 4 |
| 1110143 | 31.33 | 0.5774 | 3 |  | 32 | 1 | 3 |  | 33.67 | 0.5774 | 3 |  | 35 | 0 | 3 |
| 1110560 | 34.5 | 2.517 | 4 |  | 35 | 2.449 | 4 |  | 35.75 | 2.062 | 4 |  | 37 | 3 | 3 |
| 1110739 | 31.8 | 1.789 | 5 |  | 32.8 | 1.643 | 5 |  | 34.2 | 2.168 | 5 |  | 35 | 2.449 | 4 |
|  |  |  |  |  |  |  |  |  |  |  |  |  |  |  |  |
| Ct values for ORF1ab determined by BioGerm nucleic extraction kit and BioGerm RT-PCR kit combined with ABI 7500 PCR instrument | | | | | | | | | | | | | | | |
| 1110061 | 33.4 | 2.702 | 5 |  | 34.4 | 2.191 | 5 |  | 34.8 | 1.924 | 5 |  | 36.25 | 0.9574 | 4 |
| 1110125 | 31.67 | 0.5774 | 3 |  | 32.67 | 0.5774 | 3 |  | 33.67 | 2.517 | 3 |  | 37.75 | 2.872 | 4 |
| 110013B3 | 35 | 0.8165 | 4 |  | 35 | 1.414 | 4 |  | 36 | 1.414 | 4 |  | 37.25 | 0.9574 | 4 |
|  |  |  |  |  |  |  |  |  |  |  |  |  |  |  |  |
| Ct values for N determined by Tianlong nucleic extraction kit and BioGerm RT-PCR kit combined with Gentier PCR instrument | | | | | | | | | | | | | | | |
| 1110084 | 33.4 | 0.8944 | 5 |  | 34.8 | 1.304 | 5 |  | 35.6 | 1.14 | 5 |  | 36.75 | 0.9574 | 4 |
| 1110100 | 33.75 | 0.5 | 4 |  | 34.75 | 0.5 | 4 |  | 35.75 | 0.5 | 4 |  | 36.33 | 0.5774 | 3 |
| 1110155 | 32.67 | 1.155 | 3 |  | 33.67 | 1.155 | 3 |  | 34.67 | 0.5774 | 3 |  | 36 | 0 | 3 |
| 1111187 | 33.6 | 1.14 | 5 |  | 34.2 | 1.095 | 5 |  | 34.8 | 1.304 | 5 |  | 35.75 | 1.708 | 4 |
| 110002B6 | 33.6 | 1.14 | 5 |  | 34.6 | 0.5477 | 5 |  | 35 | 1 | 5 |  | 36 | 0.8165 | 4 |
| 110046B1 | 32.67 | 0.5774 | 3 |  | 33.33 | 0.5774 | 3 |  | 34.33 | 0.5774 | 3 |  | 34.67 | 1.155 | 3 |
|  |  |  |  |  |  |  |  |  |  |  |  |  |  |  |  |
| Ct values for N determined by Tianlong nucleic extraction kit and BioGerm RT-PCR kit combined with SLAN PCR instrument | | | | | | | | | | | | | | | |
| 1110051 | 32.67 | 0.5774 | 3 |  | 33.67 | 0.5774 | 3 |  | 34.33 | 0.5774 | 3 |  | 35.33 | 1.155 | 3 |
| 1110057 | 33.2 | 0.8367 | 5 |  | 34 | 1 | 5 |  | 35.2 | 1.095 | 5 |  | 35.5 | 0.5774 | 4 |
| 1110091 | 33 | 1.225 | 5 |  | 33.8 | 1.304 | 5 |  | 34.6 | 1.14 | 5 |  | 36 | 1.826 | 4 |
|  |  |  |  |  |  |  |  |  |  |  |  |  |  |  |  |
| Ct values for N determined by Tianlong nucleic extraction kit and BioGerm RT-PCR kit combined with ABI7500 PCR instrument | | | | | | | | | | | | | | | |
| 1110006 | 32.25 | 0.9574 | 4 |  | 33.75 | 0.9574 | 4 |  | 34.5 | 0.5774 | 4 |  | 35.33 | 1.155 | 3 |
| 1110011 | 33.67 | 0.5774 | 3 |  | 35 | 1 | 3 |  | 35.33 | 0.5774 | 3 |  | 37 | 1 | 3 |
| 1110017 | 33.6 | 1.14 | 5 |  | 34.8 | 0.4472 | 5 |  | 35.4 | 0.5477 | 5 |  | 36.5 | 0.5774 | 4 |
| 1110081 | 32.4 | 1.342 | 5 |  | 33.4 | 1.342 | 5 |  | 34.4 | 1.517 | 5 |  | 35.5 | 1 | 4 |
| 1110143 | 33 | 1 | 3 |  | 34 | 1 | 3 |  | 35 | 1 | 3 |  | 35.67 | 1.528 | 3 |
| 1110560 | 35 | 1.414 | 4 |  | 35 | 1.826 | 4 |  | 35.75 | 1.5 | 4 |  | 36.67 | 1.528 | 3 |
| 1110739 | 34 | 1.871 | 5 |  | 34.8 | 1.643 | 5 |  | 35.2 | 1.483 | 5 |  | 36.5 | 2.082 | 4 |
|  |  |  |  |  |  |  |  |  |  |  |  |  |  |  |  |
| Ct values for N determined by BioGerm nucleic extraction kit and BioGerm RT-PCR kit combined with ABI 7500 PCR instrument | | | | | | | | | | | | | | | |
| 1110061 | 34.6 | 1.817 | 5 |  | 35.6 | 2.074 | 5 |  | 35.8 | 1.304 | 5 |  | 36 | 0.8165 | 4 |
| 1110127 | 33.6 | 0.8944 | 5 |  | 34.2 | 0.4472 | 5 |  | 35.4 | 0.8944 | 5 |  | 36.75 | 0.9574 | 4 |
| 110013B3 | 35.75 | 0.9574 | 4 |  | 35.5 | 1 | 4 |  | 36.75 | 1.893 | 4 |  | 37.25 | 1.708 | 4 |

Abbreviations: SD, standard deviation; No., number; Ct, cycle threshold; Lab, laboratory.
